# Can Demographic and Exposure Characteristics Predict Levels of Social Support in Survivors from a Natural Disaster?

**DOI:** 10.1371/journal.pone.0065709

**Published:** 2013-06-11

**Authors:** Filip K. Arnberg, Lennart Melin

**Affiliations:** 1 National Centre for Disaster Psychiatry, Department of Neuroscience, Uppsala University, Uppsala, Sweden; 2 Department of Psychology, Uppsala University, Uppsala, Sweden; Tehran University of Medical Sciences, Iran (Republic of Islamic)

## Abstract

**Objective:**

Lack of social support is a strong predictor for poor mental health after disasters. Psychosocial post-disaster interventions may benefit from targeting survivors at risk of low support, yet it is unknown whether demographic and disaster exposure characteristics are associated with social support. This study assessed if age, gender, educational status, cohabitation, and disaster exposure severity predicted aspects of informal social support in a cohort of Swedish survivors from the 2004 Southeast Asian tsunami.

**Methods:**

The participants were 3,536 disaster survivors who responded to a mail survey 14 months after the disaster (49% response rate). Their perceptions of present emotional support, contact with others, tangible support, negative support and overall satisfaction with informal support were assessed with the Crisis Support Scale and analysed in five separate ordinal regressions.

**Results:**

Demographic factors and exposure severity explained variation in social supports although the effect size and predictive efficiency were modest. Cohabitation and female gender were associated with both more positive and more negative support. Single-household men were at risk for low emotional support and younger women were more likely to perceive negative support. Higher education was associated with more positive support, whereas no clear pattern was found regarding age as a predictor. Disaster exposure severity was associated with more negative support and less overall support satisfaction.

**Conclusions:**

After a disaster that entailed little disruptions to the community the associations between demographic characteristics and social support concur with findings in the general population. The findings suggest that psychosocial disaster interventions may benefit from targeting specific groups of survivors.

## Introduction

A significant proportion of survivors experience poor mental health in the aftermath of natural disasters, and reactions of posttraumatic stress (PTS) are prevalent [1]. PTS include intrusive recollections of the event; avoidance of places, persons and situations that reminds of the event; and increased arousal and wariness [2]. PTS symptoms often fade within one year after the event for the majority of survivors [3]; however, there is a substantial risk of chronic PTS that may affect the survivors for decades [4]. There is a need to understand the complex interplay of factors that influence the risk of chronic PTS.

Social relationships have long been known to be associated with health [5], [6]. Loneliness and perceptions of social isolation increase the risk for a range of adverse outcomes, including all-cause mortality [7]. Lack of social support after traumatic events is an important predictor for mental disorders [8], [9] and has shown to buffer the impact of exposure to the traumatic event [10]. Indeed, recent guidelines for psychosocial interventions after disasters emphasize the promotion and development of positive relationships to protect against the development of persistent PTS [11], [12]. A critical issue, then, is whether it is possible to predict who will benefit the most from promotion of social support among disaster survivors.

Social support refers to the functions performed for an individual by others which typically includes emotional and tangible assistance [13]. Emotional support involves expressions of love and caring, esteem and value, encouragement, and sympathy. Informational assistance is the provision of facts or advice that may help a person solve problems, and tangible or instrumental support consists of offering or supplying behavioural or material assistance with practical tasks or problems.

Disaster survivors most often use informal support [14]; that is, support provided by family, relatives, and friends. Informal social support can be divided into structural support, defined by its size and density, and functional support, defined by the quality of support [15]. Functional support, in turn, comprises both emotional support and tangible or instrumental support.

With support from friends and family, the survivors have opportunities to engage with reminders of the event, leading to the weakening of associations between contextual cues for traumatic content and feelings of extreme fear or anxiety [16]. In contrast, avoiding discussing the traumatic event with others is associated with increased PTS at a later time [17]. Although access to support depends on having structural ties to other people, perceived support has a greater influence than social network and received support on PTS [18], [19], and received support is not always beneficial [19], as it might be perceived as intrusive, of the wrong kind, and contribute to poorer self-esteem [20]. Negative support includes others’ aversive reactions to the individual’s attempts at seeking support (e.g., dismissing the survivor’s feelings or refusing to give practical assistance) but is not analogous to lack of positive social support, as these aspects repeatedly show low associations [21], [22]. Positive support has a lesser impact than negative support on PTS [8], [23], and the increased risk of PTS in women compared to men may in part be due to the women experiencing more frequent negative support [23].

The different aspects of social support reflect different underlying dimensions that show theoretical and empirical dissimilarity [8], [21], [24], [25]. Nonetheless, these aspects have often been assessed by one summary measure [8] or by a proxy such as marital status [7]. Due to their diversity and distinct effects on mental health, one may gain insight from assessing aspects of support separately [7], [8].

Little is known about whether social support after a disaster varies as a function of demographic characteristics and disaster exposure, although this association has both theoretical and practical implications. Social support after disasters has rarely been studied as a dependent variable; thus there may be unknown factors that have an effect on both support and mental health after disasters [26]. Also, psychosocial interventions after disasters could benefit from knowledge about differences in support among survivors. For example, building social capital in deprived communities have recently been suggested as a means to improve mental health [27].

Findings from various trauma samples suggest that social support differs as a function of age and gender [21], and there are age and gender differences with regard to coping with stress [28]. Lower socio-economic status and younger age are associated with loneliness [29]. In contrast, positive social support seem to be associated with younger age in the general population, as well as with female gender and high education, whereas negative support seem to be related to being male and low education [22]. Living in a single-household is associated with increased social isolation in men but not in women [30], and marriage is protective against mental health problems and suicide [31].

After a disaster, survivors of different age, gender, and marital status may perceive different levels of social support and therefore may benefit from supportive interventions to different degrees. There may also be differences in social support related to the survivors’ exposure to the disaster. Hence, the aim of the present study was to assess whether demographic and traumatic exposure characteristics are associated with aspects of informal social support after a natural disaster.

## Methods

### Ethics Statement

The study protocol was approved by the Regional Ethical Review Board in Uppsala, Sweden. All participants provided written informed consent. Participants between 16 and 18 years of age needed written consent from their caregivers. The study was conducted in accordance with the principles expressed in the Declaration of Helsinki.

### Procedure

This study was part of a follow-up of Swedish tourists afflicted by the Indian Ocean tsunami on December 26, 2004, and previous findings from the data herein have assessed the impact on mental health with regard to exposure severity [1] and social support [10]. The Swedish authorities registered 13,638 adult residents repatriated from Southeast Asia between December 27, 2004 and January 15, 2005. The 21 counties in Sweden were approached to allow for the survey to be sent to residents in their respective county. Residents in the 10 counties that agreed to participate were identified and the questionnaire was distributed in March 2006. The survey was based on a Norwegian follow-up of tsunami survivors [32] and adapted to Swedish in collaboration with the Department of Medical Epidemiology and Biostatistics and with the Center for Family and Community Medicine, Karolinska Institutet, Sweden. Only variables of relevance to the current study are reported.

### Participants

The questionnaire was sent to 10,501 individuals ≥16 years of age (77% of those registered by the authorities upon repatriation). Of 4,932 returned questionnaires there were 4,910 that could be retained for analysis. There were 385 individuals who rejected participation per e-mail or telephone, of whom 59% stated not being in an afflicted region as their reason, and 1% stated that they could not cope with filling out the questionnaire. Nonresponse was weakly associated with younger age and living in a large city (data not shown).

In order to select a sample that most likely had experienced a potentially traumatic stressor, respondents were included in this study if they had been exposed to any of a number of exposure criteria: The *direct exposure* group were those who experienced threat to life, and/or were caught by the waves, and/or were severely injured in the disaster; *indirect exposure* were those who had not experienced the above, but experienced death of, or life threat to close ones; and *vicarious exposure* were those who had not been exposed to any of the above but witnessed horrifying events (i.e., deceased people, survivors with severe injuries, people searching for others among corpses).

### Measures

The Crisis Support Scale (CSS) [33] was used to assess levels of current support in relation to the event from friends and relatives. The CSS is composed of seven items with a seven-point scale ranging from never (1) to always (7), with the items being 1. *availability of others*; 2. *contact with others with similar experiences*; 3. *confiding in others*; 4. *emotional support*; 5. *tangible support*; 6. *negative response*; and 7. *overall satisfaction with support*. Item 7 has previously been analysed separately from the summated score of the other items, with item *negative response* reversed [33]. Also, a composite score for item 1–4 has been used, with *negative response* analysed separately [23]. A factor analysis found that the CSS contained two factors where items 1, 3, and 4 loaded on one factor, *negative response* was a single item on the second factor, and *contact with others* loaded on both factors [21]. In this study, the Cronbach’s alpha for items 1–6 was.73. Items *contact with others* and *negative response* had low item-total correlations, *r* = .35 and.11, respectively. Omitting these two items increased the internal consistency for the four remaining items to Cronbach’s alpha = .83. Thus, the mean score of items 1, 3, and 4 was analysed as a composite measure of perceived *emotional support*, and the remaining items were analysed as *contact with others*, *negative support*, and overall *satisfaction with support*.

Cohabitation was coded as either living with spouse/partner or being single/widow/widower. Educational status was coded as university degree or not. Age was grouped into adolescents (16–19 years), young adults (20–29 years), middle-aged (30–49 years; reference group), aged (50–65 years), and retired (≥65 years).

### Statistical Analysis

Ordinal regressions [34] were used to estimate the associations between demographic and exposure characteristics and social support with a separate regression for each aspect of social support. The ordinal odds ratio summarises the average change in odds for moving across any binary social support threshold, and is valid over all thresholds. The assumption that odds ratios are homogenous across thresholds was satisfied by collapsing the two lowest scoring options (after reversal of negative support) into one category. In addition, an assessment of binary logistic regression odds ratios for each threshold of each dependent variable revealed small differences in direction and on average, which indicated homogenous odds ratios across thresholds. To assess joint effects of predictor variables, first-order interaction terms were added to the regression equations and subsequently removed if *p*>.05.

A sensitivity analysis was performed by duplicating the regression analyses without interaction terms on a new dataset including the original data and imputed cases. The predictors and outcomes for the set of imputed cases were given frequency distributions that reflected the distributions in the respondents. The bivariate associations between predictors and outcomes were set to be unrelated (*ρ* = 0). The odds ratios with corresponding 95% confidence intervals (CIs) are reported, together with Likelihood ratio *R* ^2^ (*R*_L_^2^) and predictive efficiency (τ_p_). The τ_p_ indicates the reduction in discrete classification error [35]. Analyses were performed with PASW Statistics 18 (SPSS Inc., Chicago, IL).

## Results

### Preliminary Analysis

Of the 4,910 respondents there were 3,536 (72%) who fulfilled the exposure criteria. There were missing data for social support, from 1% for emotional support to 4% for tangible support. An age of ≥65 years was associated with higher probability of missing values (Table 1). Forty participants (1%) had missing values for all social support items and were removed from further analyses.

**Table 1:** Demographic characteristics, disaster exposure and missing values on the Crisis Support Scale.

	Total	MissingValues^a^
Variables	N (Column %)	N (Row %)
*Gender*		
Men	1540 (44)	85 (6)
Women	1996 (56)	106 (5)
*Age Category*		
16–19	318 (9)	16 (5)
20–29	574 (16)	16 (3)
30–49	1625 (46)	68 (4)
50–64	894 (25)	71 (8)
≥65	125 (4)	20 (16)
*Education* b		
University	1501 (43)	73 (5)
Below University	2019 (57)	116 (6)
*Marital status* c		
Partner	2828 (81)	152 (5)
Single	669 (19)	33 (5)
*Exposure*		
Direct	1926 (55)	95 (5)
Indirect	655 (19)	44 (7)
Vicarious	955 (27)	52 (5)
**Total**	**3536 (100)**	**191 (5)**

a Missing ≥1 item on the Crisis Support Scale.

b Missing data *n* = 16 (0.5%).

c Missing data *n* = 39 (1.1%).

**Figure 1:**
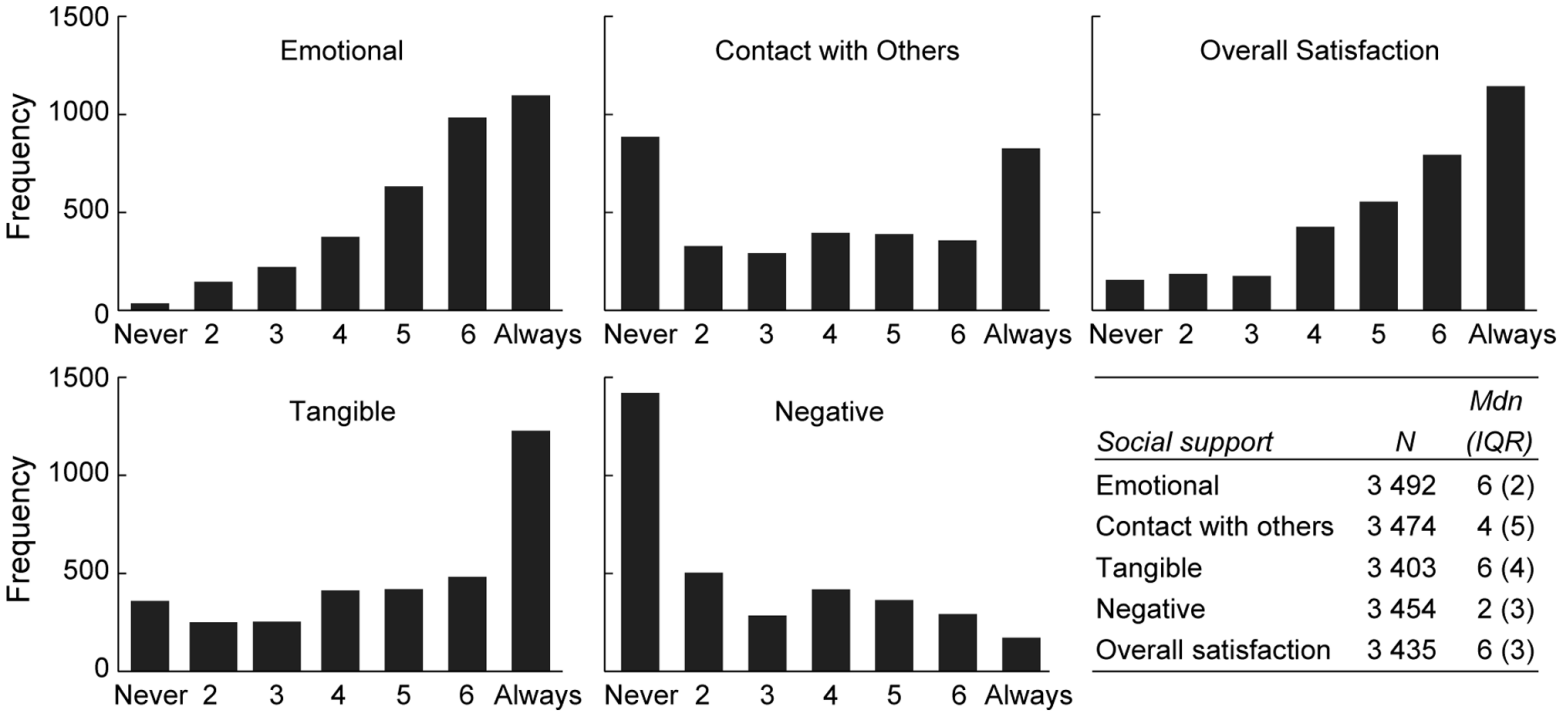
Descriptive statistics for dimensions of social support in survivors from a natural disaster.

For demographic characteristics of the sample, see Table 1. The average age was 42 years (*SD* = 14). The majority of participants (95%) had been raised in Sweden, 2% in the Nordic countries, and another 2% in the remaining European countries. There were 42% who had experienced life threat to themselves or to close relatives, 28% had been swept away by the waves, and 11% was bereaved of close friends or relatives in the disaster. The items were skewed except for contact with other survivors (Figure 1). The sum of items 1–6 with the item negative response reversed was calculated for comparison purposes; the CSS total had a mean of 30.7 (*SD* = 7.56) and a range of 6 to 42.

### Social Support and Demographics

Each social support item was the outcome variable in five separate ordinal regression models. The model fit was statistically significant for all models, indicating better fit than the intercept only model, whereas the coefficients of determination showed a very modest reduction in classification error (Table 2). The models were reanalysed adjusting for additional stressful life events after the tsunami, but this adjustment produced negligible differences in model parameters (data not shown).

**Table 2:** Ordinal regressions with demographic and disaster exposure characteristics predicting social support in survivors 14 months after a natural disaster.

				Emotional			Negative^a^			Tangible			Contact with others			Satisfaction		
Predictor		Category		OR	95% CI		OR	95% CI		OR	95% CI		OR	95% CI		OR	95% CI	
Gender		Men		**0.78**	**0.69–0.88**		**1.86**	**1.64–2.12**		**0.85**	**0.75–0.96**		0.91	0.80–1.02		**0.87**	**0.77–0.99**	
		Women		1.0	Ref.													
Cohabitation		Partner		**1.28**	**1.10–1.49**		**1.37**	**1.17–1.60**		**1.51**	**1.30–1.77**		**1.22**	**1.04–1.42**		**1.18**	**1.01–1.38**	
		Single-household		1.0	Ref.													
Education		University		**1.21**	**1.06–1.37**		1.01	0.89–1.15		**1.19**	**1.05–1.36**		**1.25**	**1.10–1.42**		**1.29**	**1.14–1.47**	
		Below University		1.0	Ref.													
Age		16–19		1.14	0.91–1.43		0.88	0.69–1.1		0.90	0.72–1.14		1.22	0.98–1.54		1.05	0.84–1.32	
		20–29		1.04	0.87–1.24		0.86	0.72–1.0		1.16	0.97–1.38		**1.34**	**1.12–1.59**		0.96	0.81–1.15	
		30–49		1.0	Ref.													
		50–64		0.96	0.83–1.11		**1.43**	**1.22–1.66**		**1.19**	**1.03–1.39**		**0.72**	**0.62–0.83**		1.08	0.93–1.26	
		≥65		1.16	0.82–1.62		**2.18**	**1.49–3.20**		0.98	0.69–1.39		0.86	0.60–1.21		1.04	0.73–1.48	
Exposure severity		Vicarious		0.97	0.84–1.11		**1.54**	**1.33–1.79**		1.11	0.96–1.28		**0.70**	**0.61–0.81**		**1.48**	**1.29–1.71**	
		Indirect		**1.19**	**1.01–1.40**		**1.25**	**1.06–1.48**		1.14	0.96–1.34		1.10	0.94–1.29		**1.40**	**1.19–1.65**	
		Direct		1.0	Ref.													
Model parameters	Model fit^b^		?^2^ = 42.8***			?^2^ = 209.9***			?^2^ = 54.7***			?^2^ = 97.7***			?^2^ = 64.3***			
	*R*_L_^2^		.004			.019			.005			.009			.006			
	τ_p_		.115			.228			.187			.170			.146			
	*N*		3 454			3 347			3 367			3 437			3 399			

The odds ratio (OR) with 95% confidence interval (CI) reflects the ratio of the odds of being in a higher category than the reference group. All statistically significant odds ratios are presented in boldface. *R*_L_^2^ = Likelihood ratio *R* ^2^.

a Negative support is reversed so that for all support items higher scores indicate more positive or less negative support.

b Likelihood ratio test, *df* = 9.

*** *p*<.001.

The differences in social support among groups were small (Table 2). Men were less likely than women to perceive both positive and negative support. Cohabiting participants were more likely to experience social support than those in a single-household. Having a university degree was associated with more positive social support, but not with lesser negative support. For age, no overall pattern emerged, although participants who were ≥50 years of age were less likely to perceive negative support than participants in the reference group. Disaster exposure was associated with lower overall support and more negative support.

**Figure 2:**
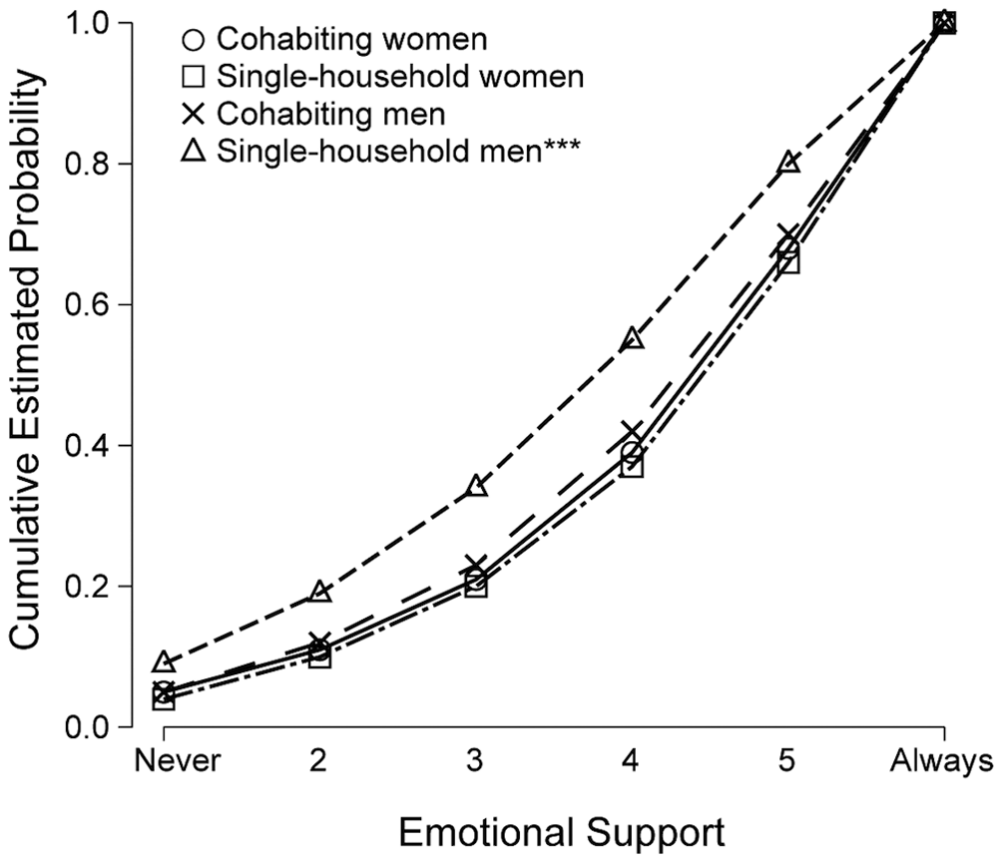
Cumulative estimated probability of emotional support by gender and cohabitation. Adjusted for age, educational status, and disaster exposure severity. ***Cohabitation × Gender interaction, *p*<.001.

**Figure 3:**
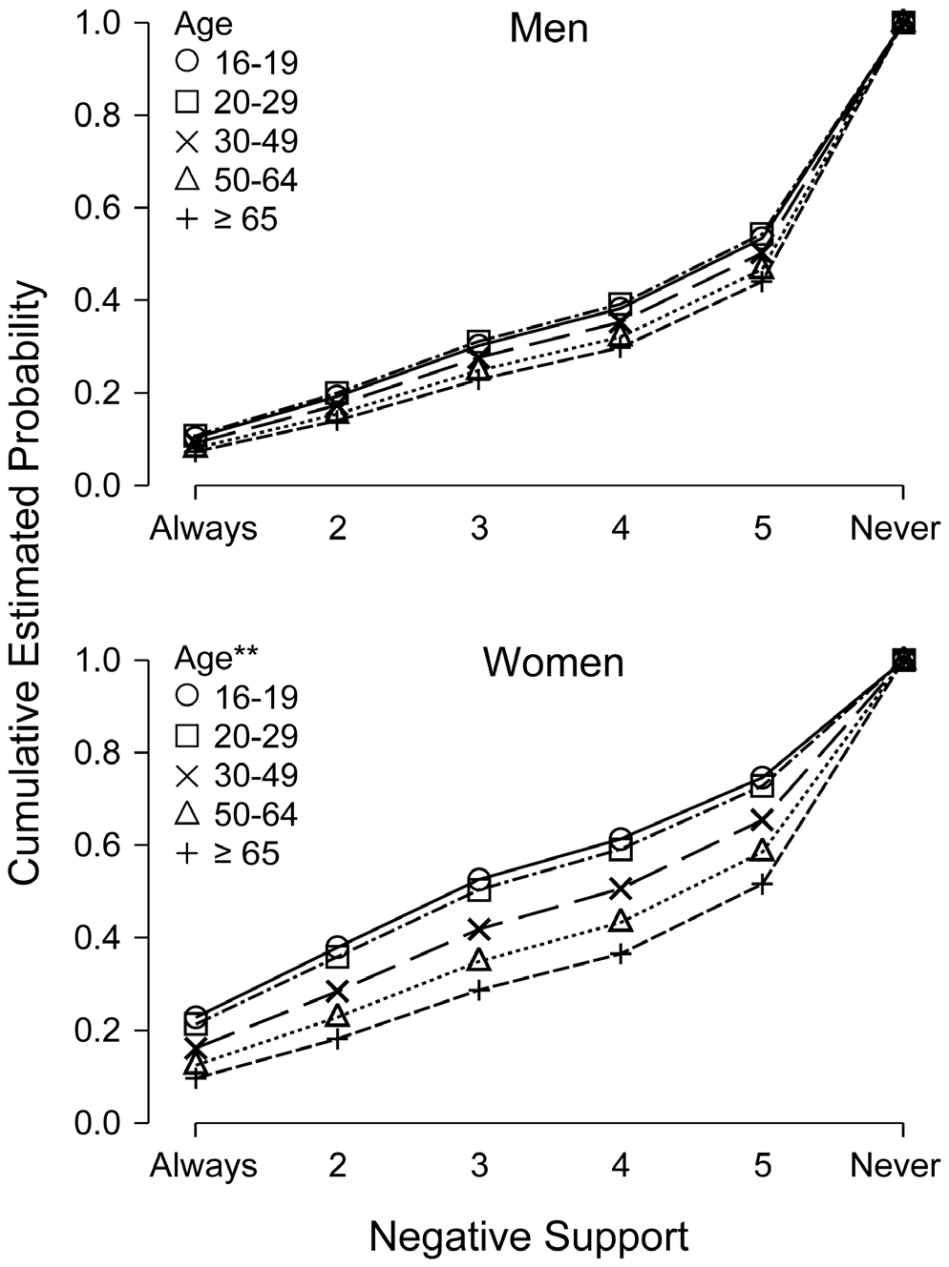
Cumulative estimated probability of negative support by gender and age. Adjusted for cohabitation, educational status, and disaster exposure severity. **Age × Gender interaction, *p*<.01.

First-order interaction terms were then examined. Interactions terms with *p*<.05 were found only in the regressions on emotional and negative support. For emotional support, only the interaction Cohabitation × Gender was statistically significant, Wald χ^2^ = 15.5, *p*<.001, suggesting that living in a single-household was associated with less emotional support for men but not for women (Figure 2). With the interaction included, the parameters and fit indicia were minimally altered (data not shown) whereas the main effect of gender was augmented, OR = 0.47, 95% CI [0.36, 0.62], *p*<.001, and the main effect of cohabitation disappeared, OR = 0.98 [0.80, 1.19], *p* = .83. For negative support, the regression coefficients for Age × Gender indicated that younger age was associated with more negative support, Wald χ^2^ = 6.86, *p* = .009. The main effects of age and gender were slightly attenuated by inclusion of the interaction term and the fit indicia were slightly improved (data not shown). The Age × Gender interaction is illustrated in Figure 3.

### Sensitivity Analysis

Based on a study of the nonrespondents in highly similar survey of tsunami registrants in a neighboring Scandinavian country [36] we assumed that 50% of the nonrespondents were eligible for this study (i.e., fulfilled the exposure criteria). Hence, we imputed a set of 2,475 cases for which the predictors were unrelated to the outcomes. Regression analyses with the original sample and the imputed nonrespondents combined showed that statistically significant odds ratios with a 95% CI within the range of 0.96–1.04 in the original analyses now became nonsignificant (i.e., indirect vs. direct exposure for emotional support; men vs. women, and 50–64 vs. 30–49 years for tangible support; partner vs. single-household for contact with others and for satisfaction with support).

## Discussion

The associations between demographic and exposure characteristics and aspects of social support were assessed in a sample of Swedish survivors 14 months after the 2004 Southeast Asia tsunami. Overall, demographic characteristics and disaster exposure were associated with social support but demonstrated modest predictive efficiency. Women were more likely to perceive both positive and negative support, which is consistent with previous findings in the general population [22], [37], [38] whereas studies on trauma samples reported no association between gender and negative support [21] and that men perceive more positive support than women [21], [39]. Norris et al. [39] proposed that women perceived lesser support than men due to women being more affected than men by the disruption of social ties in the neighbourhood. The Swedish tsunami survivors returned to mainly intact communities and therefore may be more similar to a general population regarding overall patterns and gender differences in social support.

Men in single-households are more likely to experience social isolation than are women, as men more often have their spouse as their only confidant [30]. Women are more likely to have several confidants and hence marital status would not be expected to moderate women’s perceptions of emotional support [40].

There was an inconsistent pattern of associations between age and positive social supports. Studies on the general population [22] and various trauma samples [21] have found that more positive support is associated with younger age. However, no association between age and support was found after the 1999 floods in Mexico [39]. Findings from a survey of the general population indicate that perceptions of negative support increase in individuals from 20 to 49 years whereas those 50–59 years of age perceive less negative support than any other age group [22]. This is consistent with findings in the present study, where participants above 50 years of age perceived less negative support than those aged 30–49. Interestingly, the interaction between age and gender implied that younger women, but not men, were more likely to experience negative support. The inconsistencies in the literature may result from a lack of attention towards interactions between age and gender. Having a university degree was associated with greater probability of positive support, but not with negative support, in contrast to findings that loneliness is more likely in lower socio-economic strata [29]. These differences may be explained in that loneliness may be similar to lack of positive support whereas negative support reflects a separate concept [41].

Disaster exposure demonstrated associations with overall support satisfaction and negative support, but similarly to after the 1999 floods in Mexico [39], exposure was not associated with positive support. The findings that more severely exposed survivors experience lesser satisfaction and more negative support suggest that these survivors perceived a need for more, or different, support than what was offered and warrants further study.

In summary, the findings concur more with those found in general population samples than with findings from other disasters, which concern events that have disrupted the afflicted communities. This suggests that after disasters where the disruption of communities is minimal, relief organizations and other agencies could draw from existing literature on social support in general populations to inform the targets of their interventions. Nonetheless, generalization of the findings herein is constrained by the societal context. More research is clearly needed, particularly studies relevant to low-income countries, before it is possible to reliably identify survivors who lack the support needed for successful adaptation and recovery.

### Limitations

Posttraumatic stress during the first year after the disaster could have influenced the impact of gender and disaster exposure on social support. Previous findings suggest, however, that social support influences distress up to 18 months after disaster, whereas the opposite is true 18–24 months post disaster [18]. Therefore, we chose to not control for current posttraumatic stress since it may be caused by both the predictor and outcome variables, which would increase risk for bias [42].

Moreover, the present study fails to provide conclusions as to whether groups of disaster survivors might differ in social support during the first time after the event. Governmental bodies in Sweden arranged for various psychosocial interventions during the first six months after the tsunami. The findings herein would thus reflect to some extent the effects of these interventions. However, a long-term follow-up of Vietnam veterans demonstrated that current, but not homecoming, emotional sustenance predicts both the development and the maintenance of PTSD [43]. Hence, the current findings may be relevant with respect to the maintenance of negative psychological consequences after disasters.

The sum score of CSS found in this study is comparable with findings within the same time after the event [44], [45]. Assessments of social support with single items do not necessarily result in a decreased precision in measurement [23], and analyses of aggregate measures that contain items reflecting diverse factors are not recommended [46]. Nevertheless, the measurement of social support by few or single items yields limited variation in social support that may have attenuated the differences in social support. In addition, the suboptimal distributions among items (see Figure 1) indicate that the CSS is in need of further refinement. Further studies with appropriate measures of social support may elucidate greater variation among groups of survivors.

The proportion of missing data for participants who were ≥65 years old was substantial, which raises concerns with regard to the representation particularly in this age group. Moreover, there was a low response rate. Based on other studies [36] and on the reasons given herein for not participating we assumed a 50% oversampling in the nonrespondents. The sensitivity analysis indicated that the associations found herein generally were robust even if predictors and support variables were unrelated in the group of nonrespondents. Although any sensitivity analysis of nonresponse can only assume the actual values in nonexistent data, assuming a zero association between predictors and outcomes in nonrespondents provides a fairly conservative but straightforward assessment of nonresponse bias. Based on this analysis it seems reasonable to conclude that any bias induced by nonresponse could attenuate the associations between predictors and outcomes, but would need to be very large to alter the direction of these associations.

### Conclusions

The association between demographic characteristics and social support in disaster survivors are thus far equivocal. As of yet, there are too few studies that focus on social support as an outcome variable to pinpoint the origins of differences among studies. They may reflect variations in the disaster context, such as in the disaster characteristics, the impact of the disaster on the social support systems, as well as temporal fluctuations in social support. In this study, educational status, gender, and cohabitation were associated with several dimensions of social support and could serve as proxies for targeting groups that may benefit more from promotion of social support by psychosocial interventions after disasters. No firm conclusions can be drawn from this study alone;, however, and the low predictive efficiency raises concerns about whether demographic and exposure characteristics reliably can predict support in future events. Nonetheless, the present study provides a rare attempt of examining predictors for socials support and adds to the literature a starting point from which further studies can establish how to best inform more tailored approaches to psychosocial interventions.
